# Correlation Analysis of Nasal Septum Deviation and Results of AI-Driven Automated 3D Cephalometric Analysis

**DOI:** 10.3390/jcm12206621

**Published:** 2023-10-19

**Authors:** Natalia Kazimierczak, Wojciech Kazimierczak, Zbigniew Serafin, Paweł Nowicki, Adam Lemanowicz, Katarzyna Nadolska, Joanna Janiszewska-Olszowska

**Affiliations:** 1 Kazimierczak Private Dental Practice, Dworcowa 13/u6a, 85-009 Bydgoszcz, Poland; 2 Collegium Medicum, Nicolaus Copernicus University in Torun, Jagiellońska 13-15, 85-067 Bydgoszcz, Poland; serafin@cm.umk.pl (Z.S.);; 3 Department of Interdisciplinary Dentistry, Pomeranian Medical University in Szczecin, 70-111 Szczecin, Poland; jjo@pum.edu.pl

**Keywords:** artificial intelligence, nasal septum deviation, cephalometric analysis, orthodontics

## Abstract

The nasal septum is believed to play a crucial role in the development of the craniofacial skeleton. Nasal septum deviation (NSD) is a common condition, affecting 18–65% of individuals. This study aimed to assess the prevalence of NSD and its potential association with abnormalities detected through cephalometric analysis using artificial intelligence (AI) algorithms. The study included CT scans of 120 consecutive, post-traumatic patients aged 18–30. Cephalometric analysis was performed using an AI web-based software, CephX. The automatic analysis comprised all the available cephalometric analyses. NSD was assessed using two methods: maximum deviation from an ideal non-deviated septum and septal deviation angle (SDA). The concordance of repeated manual measurements and automatic analyses was assessed. Of the 120 cases, 90 met the inclusion criteria. The AI-based cephalometric analysis provided comprehensive reports with over 100 measurements. Only the hinge axis angle (HAA) and SDA showed significant (*p* = 0.039) negative correlations. The rest of the cephalometric analyses showed no correlation with the NSD indicators. The analysis of the agreement between repeated manual measurements and automatic analyses showed good-to-excellent concordance, except in the case of two angular measurements: LI-N-B and Pr-N-A. The CephX AI platform showed high repeatability in automatic cephalometric analyses, demonstrating the reliability of the AI model for most cephalometric analyses.

## 1. Introduction

The nasal septum is a key factor in the development of the craniofacial skeleton during ontogeny [1,2,3,4,5,6]. Research conducted on animal models has shown that the mechanical forces generated by the growing nasal septum have a crucial impact on the surrounding sutural growth sites [7,8,9]. It is believed that the development of the nasal septum and the forces it exerts on surrounding tissues are responsible for the development of midface, sagittal, and vertical maxillary growth [8,10,11].

Numerous studies have demonstrated that, depending on the criteria applied and the populations examined, 18–65% of individuals exhibit nasal septum deviation (NSD), with a significantly higher prevalence observed in the European population [12,13,14,15,16]. The impact of NSD on the development of facial and cranial asymmetry remains a subject of ongoing research. Apart from the obvious correlations, such as nasal asymmetry and unilateral nasal turbinate hypertrophy [17,18,19], more distant correlations, such as facial and palatal asymmetries, have been demonstrated [20,21,22,23]. NSD and subsequent altered respiratory functions are linked to disrupted growth of the maxilla and mandible [24,25,26]. This suggests that NSD may have far-reaching effects on craniofacial development and highlights the importance of further investigation into the potential consequences of this condition. Considering the well-documented influence of the growing nasal septum on the anatomical proportions of the developing craniofacial structure, it is therefore pertinent to explore potential correlations between nasal septum abnormalities and the anatomical proportions of the craniofacial region.

Since its introduction in 1931, cephalometric analysis has been a fundamental technique used in orthodontics and craniofacial research to assess skeletal and dental relationships in the craniofacial complex [27]. It involves the use of X-ray lateral cephalograms of the head and face to obtain precise linear and angular measurements between predefined lanmarks. These measurements are then compared to established norms, allowing us to evaluate growth patterns, diagnose malocclusions, and plan orthodontic treatment. The obtained results provide valuable insights into the relationship between the maxilla, mandible, and cranial base, contributing to the diagnosis and treatment planning process. The advancement of technology has enabled the replacement of manual measurements with digital cephalometric analysis software, facilitating quicker measurements and the automatic presentation of analysis results. In recent years, there has been a growing interest in the application of artificial intelligence (AI) in the medical sciences, particularly in the field of medical imaging. This technology has seen rapid implementation in orthodontics, specifically in the analysis of X-ray images for pre-orthodontic treatment and cephalometric analysis. Manual analysis of cephalometric X-ray images is highly operator-dependent and prone to significant variability in landmark identification [28,29,30,31]. However, the results of automatic cephalometric analysis using AI have been shown to be relatively stable and repeatable compared to manual analysis. Several studies have reported the accuracy and reliability of AI software in cephalometric analysis, demonstrating potential for improving diagnostic accuracy and reducing variability and analysis time [32,33,34,35,36,37,38]. In addition to automated cephalometric analysis, AI has already proven its effectiveness in other orthodontic pre-treatment assessments. Lo Giudice et al. have demonstrated that automated segmentation of the upper airway and hard tissues (mandible) in CBCT scans, based on convolutional neural networks (CNNs), is as accurate as an experienced reader [39,40]. This efficiency can lead to improved workflow and productivity in dental practices.

The integration of AI and cone-beam computed tomography (CBCT) in dental diagnostics has paved the way for the development of AI-based programs such as WebCeph, WeDoCeph, and CephX. These programs automate the identification of anatomical measurement points, evaluate landmarks, calculate angles and distances, and generate automated analysis reports with significant findings. The primary advantage of such software is the ability to automatically perform measurements and analyses based on craniofacial CT scans, potentially reducing the need for additional cephalometric images for patients prior to orthodontic treatment. Studies conducted thus far have demonstrated a high degree of agreement between measurements and analyses performed with CephX software and those obtained through digital cephalometric analysis [35,36,41].

The first objective of our study was to perform cephalometric analysis on computed tomography (CT) scans of our sample population using AI-based algorithms. The second aim of our research was to evaluate the correlation between NSD and the results of the cephalometric analyses of our subjects.

## 2. Materials and Methods

### 2.1. Patient Selection

This study has been approved by the bioethical committee of our institution (decision reference No. KB 227/2023).

The study material comprised CT scans performed on 120 consecutive patients aged 18 to 30 admitted to the Emergency Department of our institution between 1 January 2020, and 31 December 2022. All the CT scans were performed on the same 64-slice CT scanner (Discovery 750HD; GE Health Care; Waukesha, WI, USA) using 64 × 0.625 mm collimation, 32 cm scan FOV, 260-mA tube current, 120 kVp tube voltage, 0.625 mm slice thickness, 0.8 s per gantry rotation, and a pitch of 0.531. CT scans were acquired in the range from the vertex to the lower levels of the cervical spine, covering the whole craniofacial area.

The indications for CT scans included post-traumatic assessments in patients who experienced generalized trauma or trauma to the craniofacial area.

The inclusion criteria were as follows:

1. CT scan covering the region from the chin to the vertex;
2. Age 18–30 years, to exclude multiple missing teeth and acquired craniofacial deformations from the measurements conducted;
3. Centric occlusion of the patient’s teeth.

The exclusion criteria were as follows:

1. Fractures of the craniofacial bones;
2. Severe motion artifacts;
3. >4 teeth missing per dental arch;
4. Tumors in the craniofacial area;
5. Severe metal artifacts.

### 2.2. Cephalometric Analysis

**Figure 1:**
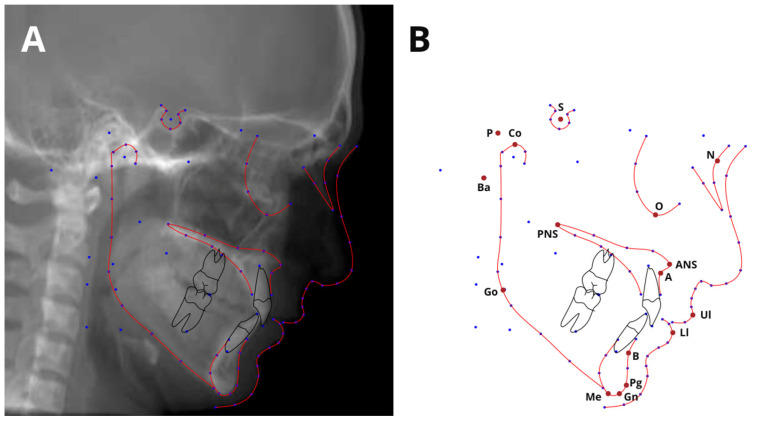
Cephalogram annotation example (**A**), showing major landmarks used in this study (**B**).

CT scans were manually uploaded into a cloud-based AI software database—CephX (ORCA Dental AI, Las Vegas, NV, USA). The software automatically performed all the available cephalometric analyses and provided a report for each patient. Supplementary Table S1 summarizes all the automatically performed analyses. The major reference points are summarized in Table 1 and presented in Figure 1.

### 2.3. NSD Analysis

**Figure 2:**
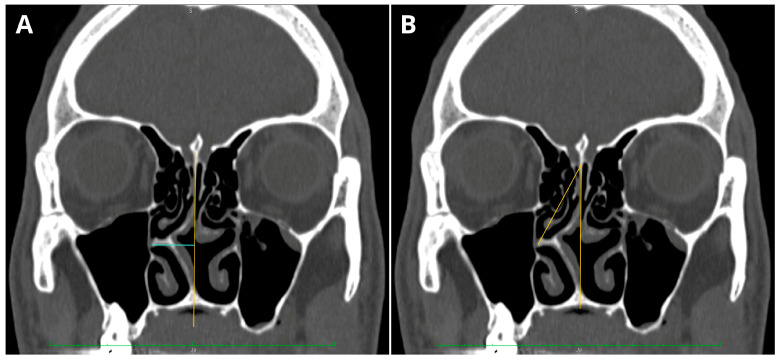
Sample of NSD measurements conducted in one patient: (**A**) perpendicular plate–vomer (PPV) measurement; (**B**) septal deviation angle (SDA) measurement.

The NSD was assessed using a method that measures the maximum deviation from an “ideal” non-deviated septum. This hypothetical, non-deviated septum was determined based on multiplanar reconstructions (MPR), drawing a straight line between the perpendicular plate of the ethmoid bone and the midline palatine suture. The deviation measurement was obtained by calculating the maximum horizontal distance from the line representing the ideal, non-deviated septum to the most outer, bony, deviated septal contour. Previous studies have demonstrated the usefulness of the perpendicular plate–vomer suture (PPV) in assessing the maximum deviation of the nasal septum in computed tomography (CT) images [5,42]. The septal deviation angle (SDA) was measured in coronal CT sections using the criteria presented in papers by Orhan and Kajan [19,43]. Figure 2 presents both types of measurements.

Due to high variability, the thickness of the nasal septum mucous membrane was not considered in the measurements. All manual measurements were performed using the OsiriX MD v. 13.1 software (Pixmeo SARL; Geneva, Switzerland).

### 2.4. Error Study

Twenty randomly selected subjects were re-examined by the same author 1 month after initial tracings. PPV and SDA measurements were repeated 1 month later by the same investigator in 20 randomly selected CT scans to calculate the intraobserver repeatability. The repeatability of measurements was assessed using a one-sided Wilcoxon test. The intraclass correlation coefficient (ICC) regarding AR calculations was calculated to assess the agreement between examinations.

Twenty randomly selected subjects were re-uploaded to the AI-software database in order to assess the repeatability of automatic measurements. The ICC regarding the results of repeated automatic analyses was calculated to assess the agreement between examinations.

### 2.5. Statistical Evaluation

Sample size was verified using an online sample size calculator (https://clincalc.com (accessed on 10 January 2023)). Clinical significance was set at the level of 1 mm in linear measurements and 1° in angular measurements. The correlations between quantitative variables were analyzed using Spearman’s correlation coefficient. The agreement between manual measurements and automatic analysis for quantitative variables was assessed using the intraclass correlation coefficient (ICC) of type 2, according to the classification by Shrout and Fleiss. The significance level for all statistical tests was set to 0.05. R 4.2.3. statistical software was used for computations.

## 3. Results

### 3.1. Population

The authors reviewed the CT scans of 120 cases. Thirty cases were excluded as they failed to meet our inclusion criteria. Measurements from 90 patients were included. The mean age of all participants was 23.9 years (SD 3.81; median 24; range 18–30). This constituted 66 males with a mean age of 23.7 (SD 3.18; range 18–30) and 24 females with a mean age of 21.15 (SD 4.03; range 18–29).

### 3.2. Automatic Cephalometric Analysis

**Figure 3:**
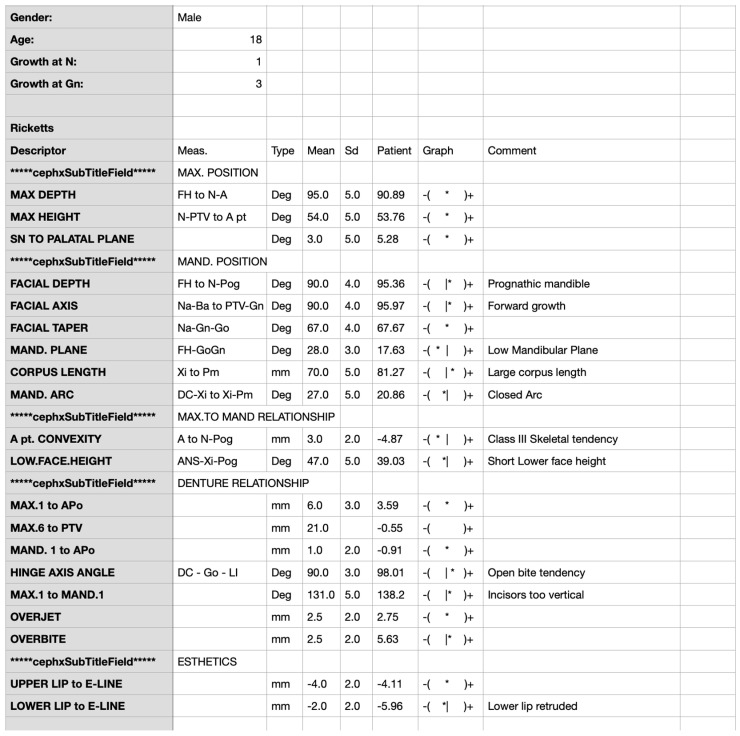
Part of sample, automatic, AI-derived cephalometric report. Patient: 18-year-old male with class III malocclusion, with graphical representations of the results regarding to norm range (column “Graph”) and clinical comments.

The results of automated cephalometric analyses conducted by AI were primarily provided in the form of reports containing >100 measurements (linear and angular), with the established range, normal values, and comments on potential clinical implications. Part of the sample report is shown in Figure 3.

### 3.3. NSD Analysis

**Figure 4:**
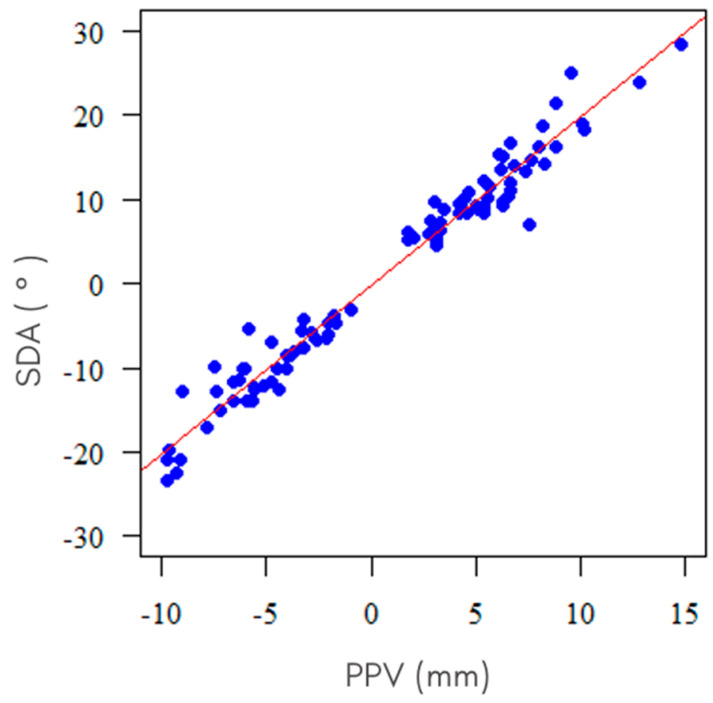
Plot displaying the correlation between SDA and PPV measurements.

The findings from the two methods employed for assessing NSD, namely SSD and PPV, demonstrated a significant (*p* < 0.001) and positive correlation, with Spearman’s correlation coefficient equating to 0.967. This suggests that as the deviation in millimeters increases, so does the angle of deviation. A graphical representation of the results from this analysis can be found in Figure 4.

**Figure 5:**
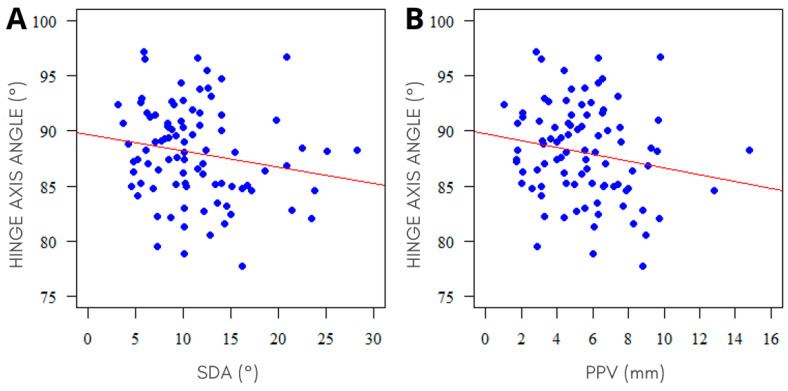
Plot displaying the correlations between HAA and NSD parameters: (**A**) SDA (r = −0.218, p = 0.039); (**B**) PPV (r = −0.186, p = 0.079).

Out of multiple correlations between NSD measurements and the results of the cephalometric analyses, only the hinge axis angle (HAA) and SDA showed a significant negative correlation (*p* ˂ 0.05, r ˂ 0). However, no significant correlation was found between HAA and PPV. See Figure 5 for a visual representation and Table 2 for the sample results of the correlation analysis.

### 3.4. Error Study

The analysis of the repeatability of PPV and SDA measurements carried out by the reader demonstrated excellent concordance (data summarized in Table 3).

The results of the repeatability of automatic cephalometric measurements showed excellent concordance in the majority of measurements. A list of parameters with poor concordance of repeated measurements is shown in Table 4. A full list of the conducted repeatability analyses is provided in the Supplementary Material—Table S2.

## 4. Discussion

Our study revealed no significant correlations between NSD parameters (SDA and PPV) and the majority of cephalometric measurements, with the exception of a weak correlation between SDA and HAA (*p* = 0.039). No significant correlation was found between PPV and HAA. The concordance of repeated manual reader measurements demonstrated excellent repeatability, while the majority of parameters in the repeated automatic analysis displayed good-to-excellent concordance, excluding two angular measurements: Lower Incisor–Nasion–B-Point (LI-N-B) and Prosthion–Nasion–A-Point (Pr-N-A). The latter indicates the need for the correction of algorithms determining single cephalometric points. Good, and in most cases excellent, concordance of the repeated analysis results indicates the high effectiveness of the tested AI algorithms in determining cephalometric landmarks.

The results of our study align with earlier published research, demonstrating the potential of AI cephalometric analysis, with varying degrees of success. A study by Lee et al. (2018) developed an AI system for automatic cephalometric landmark detection. The system was found to be highly accurate, with a mean landmark error of 1.53 ± 1.74 mm and an error of less than 2 mm for 82% of landmarks. However, the study also highlighted that the performance of the AI system varied depending on the specific landmark, suggesting the need for further refinement and training of the AI algorithm [44]. Similar results were achieved by other research teams, demonstrating a superior AI success classification rate compared to humans in some cephalometric analysis measures [30,31,45]. An interesting study by Bao et al. (2023) evaluated the accuracy of AI in the automated cephalometric analysis of reconstructed lateral cephalograms from CBCTs for 85 patients. The mean radial error for 19 chosen landmarks was 2.07 ± 1.35 mm and an error of less than 3 mm in 71.7% with the automatic program. The authors concluded that automatic analysis is almost effective enough to be acceptable in clinical work, but is not currently capable of completely replacing manual tracing [46]. Some minor inaccuracies have also been found in previously published papers regarding the reliability of CephX cephalometric analysis. Despite these issues, the authors unanimously concluded that the software is reliable for cephalometric analysis [35,36,41].

The hinge axis (HA) remains an integral part of dental assessments, especially orthognathic and orthodontic assessments, regardless of disputes over the existence of pure rotational movement in temporo-mandibular joints [47]. The HAA has emerged as a critical parameter in cephalometric analysis, providing invaluable insights into craniofacial morphology and growth patterns. The HAA refers to the angle formed by the intersection of the Frankfort horizontal (FH) plane and a line passing through the anatomical HA of the mandible [48]. The HAA in our study was defined as the angle between three cephalometric landmarks, Dc–Go–LI (distobuccal cusp of the first permanent upper molar–gonion–lower lip). Studies have shown that a larger HAA indicates a more vertical growth pattern, while a smaller HAA suggests a more horizontal growth pattern [48]. However, it is crucial to recognize that the HAA is influenced by several factors, including cranial base flexure, facial height, and the position of the mandible [48,49,50]. Our analysis revealed only a slight, negative correlation between HAA and SDA, and no correlation with PPV. This means that as the deviation angle of the nasal septum increases, the HAA decreases. Contrary to expectations, no significant correlation between HAA and PPV was found. A review of the scientific literature did not reveal any articles convincingly demonstrating a correlation between HAA and NSD. Therefore, we assume that the correlation between HAA and SDA might be coincidental. Further studies with a larger patient sample are necessary to clarify this issue.

NSD is associated with a range of direct disorders, such as headaches, rhinosinusitis, gastroesophageal reflux, and sleep apnea [51,52,53,54]. Understanding the full extent of NSD’s impact on facial and cranial asymmetry will be crucial in developing effective treatment strategies and improving patient outcomes. Among the factors influencing NSD, midfacial trauma, septal abscess, and craniofacial anomalies (e.g., cleft lip and palate) have been identified [11,23,55,56]. In some cases, a more subtle influence of the anatomical relationships of the surrounding nasofacial skeleton, including developmental constraints and morphological details of the nasal septum, has been suggested to contribute to the development of NSD [57]. A number of studies have demonstrated that a smaller volume of the nasal cavity is associated with a higher percentage of NSD, indicating a mutual influence between these phenomena [12,43,57,58]. More distant correlations between NSD and facial structures have also been found. A study published by Kim et al. [22] revealed a relationship between NSD and horizontal facial asymmetries. Additionally, Gray et al. found a high correlation between septal and palatal asymmetries and dental malocclusion [20,21,23]. A study conducted in 2015, involving 55 patients, demonstrated a correlation between NSD and asymmetries in the nasal and palatal regions [4]. However, it did not establish a correlation between these asymmetries and those of the lateral facial region.

Doubts about the generally accepted norms of cephalometric analysis results and prevailing beauty standards have been present for some time [59,60]. Additionally, the issues of beauty and attractiveness can be perceived differently by orthodontists and patients. Furthermore, apart from skeletal scaffolding, the assessment of soft facial tissues, which greatly influence the matter, is significantly limited in 2D cephalometric assessment, leading to an increasing trend of including 3D scans of the face and intraoral tissues (such as 3D dental models, 2D or 3D X-ray images, and photographs) in the standard set of examinations [60,61,62]. Such a multimodal, diverse dataset could later be used to create a more complete representation, display, and perception of the relevant structures [63]. It could also be used to create a “virtual patient” for discussing the expected treatment outcomes with the patient [64]. The issue of treatment planning, visualization of its results, and the integration of large-scale, multimodal datasets is a problem that hinders the implementation of these concepts. Therefore, there is an increasing focus on the use of AI in the analysis of 3D faces and in further treatment outcome planning [65,66,67]. Studies that assessed the accuracy of three-dimensional soft tissue prediction for Le Fort I osteotomy and orthognathic cases using Dolphin 3D software showed a limited reliability of the software [66,67]. However, in a 2022 study, Tanikawa attempted to develop AI systems that predict the 3D facial morphology after orthodontic treatment and orthognathic surgery [65]. The authors utilized lateral cephalograms, 3D facial images, and two AI systems to predict facial morphology after dental treatment. The AI systems proved to sufficiently predict facial morphology after treatment and were considered clinically acceptable. Such integration of AI, multimodal facial morphology assessment, treatment planning, and advanced visualization appears to be the future of orthodontics.

Our study evaluated lateral cephalograms, assessing the correlation of NSD mainly using vertical facial morphology. Considering the results of our work and existing data from the literature, the strongest potential connection appears to be between NSD and horizontal facial asymmetries. Taking into account the previously cited studies based on animal models, narrowing down the study group to patients with nasal septum damage at an early stage of development would likely reveal correlations not present in our group of consecutive, post-traumatic patients admitted to the Emergency Department. Furthermore, although we consider the AI-driven cephalometric analysis to be highly efficient and cost-effective in modern orthodontic practices, we believe that further studies regarding its performance must be conducted. Issues related to the influence of study quality on cephalometric analysis results, the repeatability of analysis results, and refinements and reliability of the algorithms are certainly areas that should be further explored. It should also be noted that our study is one of the initial analyses of selected morphological parameters using AI. An exciting future prospect is the ability of AI to automatically analyze large databases of imaging studies available on PACS servers at large institutions. This will certainly enable the discovery of subtle, previously unnoticed correlations between distant morphological parameters. It will undoubtedly have a significant impact on the development of fields as “hoary” and seemingly explored as anatomy.

The potential limitations of this study could be attributed to the relatively small sample size of patients included, potentially limiting the ability to detect subtle correlations between the studied parameters, which might only become apparent when analyzing larger groups. A geographic limitation was also present, as all radiographs were obtained from the same research center. Attention should also be given to the potential influence of errors in AI algorithms when determining cephalometric landmarks in the analysis results. It should also be noted that our study analyzed one of the commercially available solutions, and the obtained results should not be generalized to other AI software.

## 5. Conclusions

In conclusion, the results of the multiparametric cephalometric analysis were not correlated with the degree of nasal septum deviation in patients in our study group, except for a weak, negative correlation between HAA and SDA. The results of automatic cephalometric analyses performed by the CephX AI platform showed excellent repeatability, except for two types of angular measurements, LI-N-B and Pr-N-A, indicating the high reproductivity of the tested AI model in most cephalometric analyses.

## Figures and Tables

**Table 1:** Cephalometric landmarks used in analyses (part of).

Landmark		Definition
S	Sella	Midpoint of the sella turcica
Co	Condylion	The extreme superior point on the condylar head
ANS	Anterior Nasal Spine	Tip of the bony anterior nasal spine in the midline
N	Nasion	The most anterior point of the frontonasal suture
A	Point A	The innermost point on the contour of the maxilla between the anterior nasal spine and the alveolar crest
B	Point B	The most posterior point in the concavity along the anterior border of the symphysis
Go	Gonion	The most prominent point on the angle of the mandible formed by the junction of the ramus and the body of the mandible
Gn	Gnathion	The most inferior bony point of the mandible
Ll	Lower Lip	The most anterior point of the lower lip
Me	Menton	The most inferior point of the mandibular symphysis in the midline
N	Nasion	The most anterior point of the frontonasal suture
Or	Orbitale	The lowest point on the inferior margin of the orbit
P	Porion	The central point on the upper margin of the external auditory meatus
Pr	Prosthion	The point of alveolar contact with the upper central incisor
Pg	Pogonion	The most anterior point on the contour of the bony chin

**Table 2:** Sample of correlation analysis performed: NSD/HAA, and NSD/results of Björk–Jarabak cephalometric analysis.

Parameter	SDA (°)	PPV (mm)
	Spearman’s Rank Correlation Coefficient	Spearman’s Rank Correlation Coefficient
HINGE AXIS ANGLE (°)	r= −0.218, *p* = 0.039 *	r = −0.186, *p* = 0.079
Björk–Jarabak cephalometric analysis		
SADDLE ANGLE (°)	r = 0.017, *p* = 0.875	r = −0.02, *p* = 0.853
ARTICULAR ANGLE (°)	r = 0.103, *p* = 0.335	r = 0.104, *p* = 0.329
GONIAL ANGLE (°)	r = −0.154, *p* = 0.148	r = −0.129, *p* = 0.226
SUM OF ANGLES (°)	r = 0.044, *p* = 0.679	r = 0.03. *p* = 0.782
UPPER GONIAL ANGLE (°)	r = −0.175, *p* = 0,099	r = −0.183, *p* = 0.084
LOWER GONIAL ANGLE (°)	r = −0.002, *p* = 0.984	r = 0.023, *p* = 0.826
ANT. CRANIAL BASE (mm)	r = −0.011, *p* = 0.915	r = 0.018, *p* = 0.67
POST. CRANIAL BASE (mm)	r = 0.018, *p* = 0.866	r = 0.052, *p* = 0.626
RAMUS HEIGHT (mm)	r = 0.024, *p* = 0.825	r = 0.099, *p* = 0.353
MANDIBULAR BODY (mm)	r = −0.02, *p* = 0.851	r = −0.004, *p* = 0.972
POST. FACE HEIGHT (mm)	r = 0.031, *p* = 0.77	r = 0.096, *p* = 0.368
ANT. FACE HEIGHT (mm)	r = −0.016, *p* = 0.879	r = 0.043, *p* = 0.69
PFH:AFH (%)	r = 0.023, *p* = 0.832	r = 0.053, *p* = 0.623
ACB:MAND.BODY (%)	r = 0.004, *p* = 0.97	r = −0.023, *p* = 0.832
UI to SN (°)	r = −0.096, *p* = 0.37	r = −0.094, *p* = 0.376
UI to FH (°)	r = −0.073, *p* = 0.497	r = −0.074, *p* = 0.486
UPPER FACE HEIGHT (%)	r = −0.127, *p* = 0.233	r = −0.057, *p* = 0.596
LOWER FACE HEIGHT (%)	r = 0.125, *p* = 0.24	r = 0.065, *p* = 0.544

*—statistically significant relationship (*p* < 0.05). Abbreviations: SDA—septal deviation angle; PPV—perpendicular plate–vomer measurement.

**Table 3:** Results of repeatability of manual measurements.

Parameter	Measurement I (Mean ± SD)	Measurement II (Mean ± SD)	ICC	95% CI		Agreement (Cicchetti)	Agreement (Koo and Li)
PPV (mm)	5.19 ± 2.6	5.28 ± 2.49	0.974	0.937	0.990	Excellent	PPV (mm)
SDA (°)	10.78 ± 5.69	10.85 ± 5.73	0.972	0.931	0.989	Excellent	SDA (°)

Abbreviations: PPV—perpendicular plate–vomer measurement; SDA—septal deviation angle.

**Table 4:** List of parameters with low agreement in repeated measurements.

Parameter	Measurement 1 (Mean ± SD)	Measurement 2 (Mean ± SD)	ICC	95% CI		Agreement (Cicchetti)	Agreement (Koo and Li)
LI-N-B	21.73 ± 8.41	36.67 ± 45.54	0.000	−0.536	0.548	Poor	Poor
Pr-N-A	1.76 ± 0.66	1.85 ± 0.54	0.302	−0.287	0.730	Poor	Poor

Abbreviations: SD—Standard Deviation; I—Incisor; NB—Nasion–B-Point; Pr—Prosthion; A—A-Point; ICC—Interclass Correlation Coefficient; CI—Confidence Interval.

## Data Availability

Data are available on request.

## Supplementary Materials

The following supporting information can be downloaded at: https://www.mdpi.com/article/10.3390/jcm12206621/s1; Table S1: List of performed automatic cephalometric analyses; Table S2: Results of repeatability analysis of automatic AI cephalometric analyses.
