# Supplementary material for: Correlation Analysis of Nasal Septum Deviation and Results of AI-Driven Automated 3D Cephalometric Analysis
Source: J Clin Med. 2023 Oct 19;12(20):6621. doi: 10.3390/jcm12206621 (PMC10607148; doi:10.3390/jcm12206621)
Supplement: Supplementary file 1 [file jcm-12-06621-s001.zip › Table S1.pdf]

**Table S1**. List of performed automatic cephalometric analyses.

| Cephalometric Analysis |
|---|
| Bjork - Jarabak |
| Burlington |
| Dental Upper & Lower |
| Downs |
| Occlusal Plane |
| Biodynamic |
| McNamara |
| Owen Block |
| P.O.S. |
| Projected Growth |
| V.T.O Holdaway |
| Ricketts |
| Skeletal Antpost |
| Skeletal Vertical |
| SLU |
| Soft Tissue |
| Steiner |
| Tweed |
| Wits |
| Ricketts LS |
| Steiner LS |
| Tweed LS |
| Wits LS |
| SLU LS |
| Downs LS |
| Jarabak LS |
| Traced Ceph |
| ABO |
| Airway |
| Bimler LS |
| Burstone |
| Clark |
| COGS |
| DentalSmart |
| DiPaulo |
| Dr. Mike Zeichner |
| Eastman |
| Harvold |
| Heb Uni. |

| |
|---|
| Holdaway Graphics (IL) |
| IBO |
| IL (modified) |
| Jarabak graphics (IL) |
| JeffersonNew |
| Mahony |
| McGann |
| McGann LS |
| MCLaughin |
| McNamara graphics (IL) |
| MidContinent |
| Modified Steiner |
| Orca Free |
| Profilogram SM |
| Ricketts (IL) |
| Rondeau |
| Rondeau Modified |
| Sassouni |
| Sassouni LS / Plus |
| Steiner Tweed Graphics (IL) |
| TAU |
| Tetragon plus |
| UNC-Blacker II |
| USDI LS |
| USP |
