# Supplementary material for: Correlation Analysis of Nasal Septum Deviation and Results of AI-Driven Automated 3D Cephalometric Analysis
Source: J Clin Med. 2023 Oct 19;12(20):6621. doi: 10.3390/jcm12206621 (PMC10607148; doi:10.3390/jcm12206621)
Supplement: Supplementary file 1 [file jcm-12-06621-s001.zip › Table S2.pdf]

1 Bjork - Jarabak

Parameter	Measurement 1 (mean±SD)	Measurement 2 (mean±SD)	ICC	95% CI	Agreement (Cicchetti)	Agreement (Koo & Li)
SADDLE ANGLE (Deg)	123,77±3,4	123,92±3,71	0,967	0,895 0,990	Excellent	Excellent
ARTICULAR ANGLE (Deg)	144,73±5,17	144,55±5,65	0,955	0,858 0,987	Excellent	Excellent
GONIAL ANGLE (Deg)	125,61±4,8	125,48±5,2	0,945	0,828 0,984	Excellent	Excellent
SUM OF ANGLES (Deg)	394,11±7,67	393,94±7,8	0,992	0,972 0,998	Excellent	Excellent
UPPER GONIAL ANGLE (Deg)	52,07±3,84	51,94±4	0,946	0,831 0,984	Excellent	Excellent
LOWER GONIAL ANGLE (Deg)	73,54±6,68	73,53±6,71	0,997	0,991 0,999	Excellent	Excellent
ANT. CRANIAL BASE (mm)	68,34±12,44	68,61±12,4	0,902	0,706 0,970	Excellent	Excellent
POST. CRANIAL BASE (mm)	35,38±7,29	35,88±7,87	0,909	0,727 0,973	Excellent	Excellent
RAMUS HEIGHT (mm)	42,31±8,8	42,75±7,67	0,837	0,544 0,949	Excellent	Good
MANDIBULAR BODY (mm)	74,77±15,29	75,29±15,55	0,897	0,696 0,969	Excellent	Good
POST. FACE HEIGHT (mm)	73,98±14,68	74,84±14,3	0,869	0,621 0,960	Excellent	Good
ANT. FACE HEIGHTL (mm)	115,76±21,33	116,68±20,6	0,893	0,684 0,968	Excellent	Good
PFH:AFH (%)	63,45±4,28	63,69±4,4	0,958	0,867 0,988	Excellent	Excellent
ACB:MAND.BODY (%)	0,91±0,06	0,91±0,09	0,850	0,576 0,954	Excellent	Good
UI to SN (Deg)	101,44±5,98	101,83±5,33	0,963	0,881 0,989	Excellent	Excellent
UI to FH (Deg)	110±4,8	110,55±4,08	0,923	0,764 0,977	Excellent	Excellent
UPPER FACE HEIGHT (%)	45,08±2,02	45±1,95	0,989	0,965 0,997	Excellent	Excellent
LOWER FACE HEIGHT (%)	55,89±2,13	55,85±2,13	0,999	0,995 1,000	Excellent	Excellent

2 Dental Lower

Parameter	Measurement 1 (mean±SD)	Measurement 2 (mean±SD)	ICC	95% CI		Agreement (Cicchetti)	Agreement (Koo & Li)
LOWER I TO NB (Deg)	21,73±8,41	36,67±45,54	0,000	-0,536	0,548	Poor	Poor
LOWER I TO NB (mm)	3,86±3,36	3,74±3,39	0,998	0,991	0,999	Excellent	Excellent
LOWER I TO A/P LINE (mm)	0,18±2,45	0,08±2,58	0,985	0,951	0,996	Excellent	Excellent
LOWER I TO GOGN (Deg)	87,71±7,77	87,6±9,68	0,951	0,846	0,985	Excellent	Excellent
Interincisal Angle (Deg)	134,63±10,71	134,45±11,9	0,952	0,848	0,986	Excellent	Excellent

3 Dental Upper

Parameter	Measurement 1 (mean±SD)	Measurement 2 (mean±SD)	ICC	95% CI		Agreement (Cicchetti)	Agreement (Koo & Li)
UPPER I TO SN (Deg)	101,44±5,98	101,83±5,33	0,963	0,881	0,989	Excellent	Excellent
UPPER I TO FRANKFORT (Deg)	110±4,8	110,55±4,08	0,923	0,764	0,977	Excellent	Excellent
UPPER I TO PAL/PL (Deg)	110,03±4,28	110,25±4,08	0,970	0,905	0,991	Excellent	Excellent
UPPER I TO NA ANGLE (Deg)	18,65±4,82	18,9±3,88	0,925	0,772	0,978	Excellent	Excellent
UPPER I TO A/P LINE (mm)	5,78±3,16	5,76±2,85	0,979	0,932	0,994	Excellent	Excellent
UPPER I TO NA (MM) (mm)	2,51±2,08	2,5±1,69	0,953	0,851	0,986	Excellent	Excellent

4 Downs - SKELETAL

Parameter	Measurement 1 (mean±SD)	Measurement 2 (mean±SD)	ICC	95% CI		Agreement (Cicchetti)	Agreement (Koo & Li)
FACIAL ANGLE (Deg)	87,18±3,18	87,46±3,24	0,982	0,936	0,995	Excellent	Excellent
ANGLE CONVEXITY (DOWNS) (Deg)	172,18±7,9	172,09±8,03	0,999	0,996	1,000	Excellent	Excellent
A-B PLANE (Deg)	7,55±2,79	7,53±2,73	0,998	0,993	0,999	Excellent	Excellent
MAND. PLANE (Deg)	25,56±6,19	25,24±6,48	0,971	0,908	0,992	Excellent	Excellent
Y AXIS (Deg)	60,48±4,03	60,39±4,07	0,987	0,957	0,996	Excellent	Excellent

5 **Downs - DENTAL**

Parameter	Measurement 1 (mean±SD)	Measurement 2 (mean±SD)	ICC	95% CI		Agreement (Cicchetti)	Agreement (Koo & Li)
OCCLUSAL PLANE (Deg)	20,25±49,62	20,3±49,66	0,999	0,997	1,000	Excellent	Excellent
UI to LI (Deg)	134,63±10,71	134,45±11,9	0,952	0,848	0,986	Excellent	Excellent
LI to Occ PL. (Deg)	69,89±10,12	70,31±12,43	0,966	0,890	0,990	Excellent	Excellent
LI to MAND (Deg)	87,71±7,77	87,6±9,68	0,951	0,846	0,985	Excellent	Excellent
UI to A-Pog (mm)	5,78±3,16	5,76±2,85	0,979	0,932	0,994	Excellent	Excellent

6 **Biodynamic - BASAL - Cranial Base Form**

Parameter	Measurement 1 (mean±SD)	Measurement 2 (mean±SD)	ICC	95% CI		Agreement (Cicchetti)	Agreement (Koo & Li)
SN-BASION (Deg)	129,24±4,6	129,04±4,66	0,983	0,944	0,995	Excellent	Excellent
SN-AR (Deg)	123,77±3,4	123,92±3,71	0,967	0,895	0,990	Excellent	Excellent

7 **Biodynamic - SAGITTAL - Jaw Position**

Parameter	Measurement 1 (mean±SD)	Measurement 2 (mean±SD)	ICC	95% CI		Agreement (Cicchetti)	Agreement (Koo & Li)
SNA (Deg)	82,79±2,42	82,93±2,51	0,922	0,763	0,977	Excellent	Excellent
SN-Pg (Deg)	78,63±4,46	78,74±4,84	0,973	0,912	0,992	Excellent	Excellent
AN-Pg (Deg)	4,16±3,62	4,18±3,67	0,999	0,997	1,000	Excellent	Excellent

8 **Biodynamic - SAGITTAL - Apical Base Position**

Parameter	Measurement 1 (mean±SD)	Measurement 2 (mean±SD)	ICC	95% CI		Agreement (Cicchetti)	Agreement (Koo & Li)
SNA (Deg)	82,79±2,42	82,93±2,51	0,922	0,763	0,977	Excellent	Excellent
SNB (Deg)	77,81±3,81	77,96±4,12	0,967	0,895	0,990	Excellent	Excellent
ANB (Deg)	4,97±2,84	4,96±2,84	0,999	0,997	1,000	Excellent	Excellent

9 **Biodynamic - SAGITTAL - Dento-Basal Relations**

Parameter	Measurement 1 (mean±SD)	Measurement 2 (mean±SD)	ICC	95% CI		Agreement (Cicchetti)	Agreement (Koo & Li)
Pr-N-A (Deg)	1,76±0,66	1,85±0,54	0,302	-0,287	0,730	Poor	Poor
UI-PAL.PLANE (Deg)	110,03±4,28	110,25±4,08	0,970	0,905	0,991	Excellent	Excellent
UI-NA (mm)	2,51±2,08	2,5±1,69	0,953	0,851	0,986	Excellent	Excellent
GoGn-Cl (Deg)	68,28±4,56	68,62±6,54	0,892	0,680	0,967	Excellent	Good
LI-GoGn (Deg)	87,71±7,77	87,6±9,68	0,951	0,846	0,985	Excellent	Excellent
LI-NB (mm)	3,86±3,36	3,74±3,39	0,998	0,991	0,999	Excellent	Excellent
Pg-NB (mm)	1,82±1,75	1,82±1,82	0,991	0,971	0,997	Excellent	Excellent

10 Biodynamic - VERTICAL - Jaw Position

Parameter	Measurement 1 (mean±SD)	Measurement 2 (mean±SD)	ICC	95% CI		Agreement (Cicchetti)	Agreement (Koo & Li)
SN-GoGn (Deg)	36,2±7,84	36,1±7,9	0,992	0,975	0,998	Excellent	Excellent
Pal-Mand Angle (Deg)	27,62±6,15	27,68±6,35	0,992	0,972	0,998	Excellent	Excellent
SN-PAL.PLANE (Deg)	8,58±3,12	8,42±3	0,970	0,904	0,991	Excellent	Excellent

11 Biodynamic - DENTAL - BASAL RELATIONS

Parameter	Measurement 1 (mean±SD)	Measurement 2 (mean±SD)	ICC	95% CI		Agreement (Cicchetti)	Agreement (Koo & Li)
Mx Base-Occ Pl. (PP-OP) (Deg)	20,22±50,04	20,6±50,01	0,999	0,997	1,000	Excellent	Excellent
Mand Pl. to Occ Pl. (Deg)	22,39±5,68	22,08±6,26	0,974	0,916	0,992	Excellent	Excellent

12 McNamara

Parameter	Measurement 1 (mean±SD)	Measurement 2 (mean±SD)	ICC	95% CI		Agreement (Cicchetti)	Agreement (Koo & Li)
MAXILLARY SKELETAL (mm)	1,25±2,07	1,53±1,87	0,933	0,790	0,980	Excellent	Excellent
MAXILLARY DENTAL (mm)	2,51±2,08	2,5±1,69	0,953	0,851	0,986	Excellent	Excellent
MANDIBULAR SKELETAL (mm)	-5,48±6,74	-4,99±6,87	0,986	0,951	0,996	Excellent	Excellent

Parameter	Measurement 1 (mean±SD)	Measurement 2 (mean±SD)	ICC	95% CI	Agreement (Cicchetti)	Agreement (Koo & Li)
MANDIBULAR DENTAL (mm)	0,18±2,45	0,08±2,58	0,985	0,951 0,996	Excellent	Excellent
EFF.MAND.LENGTH (mm)	106,64±21,05	107,19±20,31	0,897	0,694 0,969	Excellent	Good
EFF.MIDFACIAL.LENGTH (mm)	82,22±15,02	82,84±14,53	0,899	0,699 0,969	Excellent	Good
MAND - MAX DIFF (mm)	24,42±7,38	24,34±7,5	0,925	0,771 0,978	Excellent	Excellent
LOW.ANT.FACE.HEIGHT (mm)	66,46±12,69	66,84±11,78	0,897	0,695 0,969	Excellent	Good
MAND PLANE ANGLE (Deg)	25,56±6,19	25,24±6,48	0,971	0,908 0,992	Excellent	Excellent
GROWTH AXIS (Deg)	86,64±5,75	86,49±5,68	0,998	0,993 0,999	Excellent	Excellent

13 Ricketts - MAX. POSITION

Parameter	Measurement 1 (mean±SD)	Measurement 2 (mean±SD)	ICC	95% CI	Agreement (Cicchetti)	Agreement (Koo & Li)
MAX DEPTH (Deg)	91,34±2,01	91,65±1,88	0,955	0,830 0,987	Excellent	Excellent
MAX HEIGHT (Deg)	57,44±3,45	57,53±3,81	0,980	0,935 0,994	Excellent	Excellent
SN TO PALATAL PLANE (Deg)	8,58±3,12	8,42±3	0,970	0,904 0,991	Excellent	Excellent

14 Ricketts - MAND. POSITION

Parameter	Measurement 1 (mean±SD)	Measurement 2 (mean±SD)	ICC	95% CI	Agreement (Cicchetti)	Agreement (Koo & Li)
FACIAL DEPTH (Deg)	87,18±3,18	87,46±3,24	0,982	0,936 0,995	Excellent	Excellent
FACIAL AXIS (Deg)	86,64±5,75	86,49±5,68	0,998	0,993 0,999	Excellent	Excellent
FACIAL TAPER (Deg)	68,35±4,03	68,46±4,78	0,938	0,809 0,982	Excellent	Excellent
MAND. PLANE (Deg)	25,56±6,19	25,24±6,48	0,971	0,908 0,992	Excellent	Excellent
CORPUS LENGTH (mm)	74,38±14,77	74,89±13,6	0,868	0,619 0,959	Excellent	Good
MAND. ARC (Deg)	26,54±5,92	25,89±4,99	0,905	0,716 0,971	Excellent	Excellent

15 Ricketts - MAX.TO MAND RELATIONSHIP

Parameter	Measurement 1 (mean±SD)	Measurement 2 (mean±SD)	ICC	95% CI		Agreement (Cicchetti)	Agreement (Koo & Li)
A pt. CONVEXITY (mm)	3,95±3,6	3,96±3,62	0,998	0,994	1,000	Excellent	Excellent
LOW.FACE.HEIGHT (Deg)	42,61±5,66	42,55±5,72	0,996	0,987	0,999	Excellent	Excellent

16 Ricketts - DENTURE RELATIONSHIP

Parameter	Measurement 1 (mean±SD)	Measurement 2 (mean±SD)	ICC	95% CI		Agreement (Cicchetti)	Agreement (Koo & Li)
MAX.1 to APo (mm)	5,78±3,16	5,76±2,85	0,979	0,932	0,994	Excellent	Excellent
MAX.6 to PTV (mm)	12,31±9,27	13,09±8,97	0,965	0,889	0,990	Excellent	Excellent
MAND. 1 to APo (mm)	0,18±2,45	0,08±2,58	0,985	0,951	0,996	Excellent	Excellent
HINGE AXIS ANGLE (Deg)	88,32±3,3	88,1±3,28	0,874	0,635	0,962	Excellent	Good
MAX.1 to MAND.1 (Deg)	134,63±10,71	134,45±11,9	0,952	0,848	0,986	Excellent	Excellent
OVERJET (mm)	4,88±2,57	4,93±2,41	0,991	0,972	0,997	Excellent	Excellent
OVERBITE (mm)	3,85±3,09	4,04±3,29	0,857	0,592	0,956	Excellent	Good

17 Ricketts - ESTHETICS

Parameter	Measurement 1 (mean±SD)	Measurement 2 (mean±SD)	ICC	95% CI		Agreement (Cicchetti)	Agreement (Koo & Li)
UPPER LIP to E-LINE (mm)	-3,41±3,7	-3,39±3,77	0,990	0,969	0,997	Excellent	Excellent
LOWER LIP to E-LINE (mm)	-4±3,57	-3,92±3,72	0,989	0,965	0,997	Excellent	Excellent

18 Skeletal Antpost

Parameter	Measurement 1 (mean±SD)	Measurement 2 (mean±SD)	ICC	95% CI		Agreement (Cicchetti)	Agreement (Koo & Li)
SNA (Deg)	82,79±2,42	82,93±2,51	0,922	0,763	0,977	Excellent	Excellent
SNB (Deg)	77,81±3,81	77,96±4,12	0,967	0,895	0,990	Excellent	Excellent
ANB (Deg)	4,97±2,84	4,96±2,84	0,999	0,997	1,000	Excellent	Excellent

Parameter	Measurement 1 (mean±SD)	Measurement 2 (mean±SD)	ICC	95% CI		Agreement (Cicchetti)	Agreement (Koo & Li)
POGONION (mm)	1,82±1,75	1,82±1,82	0,991	0,971	0,997	Excellent	Excellent
ANGLE CONVEXITY (DOWNS) (Deg)	172,18±7,9	172,09±8,03	0,999	0,996	1,000	Excellent	Excellent
WITS (mm)	3,72±3,99	3,53±4,78	0,944	0,826	0,983	Excellent	Excellent

19 Skeletal Vertical

Parameter	Measurement 1 (mean±SD)	Measurement 2 (mean±SD)	ICC	95% CI		Agreement (Cicchetti)	Agreement (Koo & Li)
GOGN-SN (Deg)	36,2±7,84	36,1±7,9	0,992	0,975	0,998	Excellent	Excellent
OM ANGLE (SCHUDY) (Deg)	22,39±5,68	22,08±6,26	0,974	0,916	0,992	Excellent	Excellent
Y-AXIS(JARABAK) (Deg)	69,04±5,57	69,1±5,59	0,997	0,989	0,999	Excellent	Excellent
Y-AXIS(DOWNS) (Deg)	60,48±4,03	60,39±4,07	0,987	0,957	0,996	Excellent	Excellent
JARABAK HEIGHT S-GO/N-ME (%)	63,45±4,28	63,69±4,4	0,958	0,867	0,988	Excellent	Excellent
PL-MAND PLANE (Deg)	27,62±6,15	27,68±6,35	0,992	0,972	0,998	Excellent	Excellent
ANT FACIAL HT(N-ANS-ME) (%)	45,08±2,02	45±1,95	0,989	0,965	0,997	Excellent	Excellent

20 Soft Tissue

Parameter	Measurement 1 (mean±SD)	Measurement 2 (mean±SD)	ICC	95% CI		Agreement (Cicchetti)	Agreement (Koo & Li)
A ANGLE (Deg)	89,17±3,08	89,4±3,17	0,982	0,941	0,995	Excellent	Excellent
NOSE (mm)	15,19±7,64	15,44±7,44	0,963	0,882	0,989	Excellent	Excellent
U/LIP : VERT (mm)	1,81±1,11	1,96±1,03	0,915	0,742	0,974	Excellent	Excellent
U/LIP : EMBR (mm)	5,11±1,62	5,3±1,63	0,931	0,788	0,979	Excellent	Excellent
U/LIP A WIDTH (mm)	14,97±2,68	15,05±2,64	0,882	0,657	0,964	Excellent	Good
U/LIP VERMILLION / WIDTH (mm)	14,66±3,75	14,82±4	0,940	0,815	0,982	Excellent	Excellent
U/LIP TAPER / STRAIN (mm)	0,31±2,91	0,23±2,75	0,973	0,912	0,992	Excellent	Excellent
A/PT CNVXTY (mm)	3,95±3,6	3,96±3,62	0,998	0,994	1,000	Excellent	Excellent

Parameter	Measurement 1 (mean±SD)	Measurement 2 (mean±SD)	ICC	95% CI		Agreement (Cicchetti)	Agreement (Koo & Li)
H ANGLE (mm)	17,44±5,06	17,65±5,53	0,983	0,944	0,995	Excellent	Excellent
L/LIP PROTRUSION (mm)	1,57±1,96	1,55±1,94	0,983	0,944	0,995	Excellent	Excellent
L/LIP EMBR (mm)	5,69±1,86	5,74±2,04	0,935	0,798	0,980	Excellent	Excellent
SOFT POG (mm)	11,93±2,78	11,85±2,43	0,944	0,825	0,983	Excellent	Excellent

21 SLU

Parameter	Measurement 1 (mean±SD)	Measurement 2 (mean±SD)	ICC	95% CI		Agreement (Cicchetti)	Agreement (Koo & Li)
SNA (Deg)	82,79±2,42	82,93±2,51	0,922	0,763	0,977	Excellent	Excellent
PNS-A (Deg)	49,2±8,75	49,56±8,26	0,899	0,701	0,970	Excellent	Good
PTV-6 (mm)	12,31±9,27	13,09±8,97	0,965	0,889	0,990	Excellent	Excellent
UI-SN (Deg)	101,44±5,98	101,83±5,33	0,963	0,881	0,989	Excellent	Excellent
UI-NA (Deg)	18,65±4,82	18,9±3,88	0,925	0,772	0,978	Excellent	Excellent
UI-NA (mm)	2,51±2,08	2,5±1,69	0,953	0,851	0,986	Excellent	Excellent
SNB (Deg)	77,81±3,81	77,96±4,12	0,967	0,895	0,990	Excellent	Excellent
NS-Gn (Deg)	69,04±5,57	69,1±5,59	0,997	0,989	0,999	Excellent	Excellent
S-Ar (mm)	35,38±7,29	35,88±7,87	0,909	0,727	0,973	Excellent	Excellent
AR-Gn (mm)	105,1±20,91	105,89±20,12	0,896	0,692	0,969	Excellent	Good
POG-NS (mm)	1,82±1,75	1,82±1,82	0,991	0,971	0,997	Excellent	Excellent
IMPA (Ang)	87,71±7,77	87,6±9,68	0,951	0,846	0,985	Excellent	Excellent
LI-APOG (mm)	0,18±2,45	0,08±2,58	0,985	0,951	0,996	Excellent	Excellent
LI-NB (Ang)	21,73±8,41	36,67±45,54	0,000	-0,536	0,548	Poor	Poor
LI-NB (mm)	3,86±3,36	3,74±3,39	0,998	0,991	0,999	Excellent	Excellent
FMIA (Ang)	64,63±10,3	65±11,1	0,965	0,888	0,990	Excellent	Excellent
LI/UI (Deg)	134,63±10,71	134,45±11,9	0,952	0,848	0,986	Excellent	Excellent
ANB (Ang)	4,97±2,84	4,96±2,84	0,999	0,997	1,000	Excellent	Excellent
WITS (mm)	3,72±3,99	3,53±4,78	0,944	0,826	0,983	Excellent	Excellent
SN-PAL.PLANE (Deg)	8,58±3,12	8,42±3	0,970	0,904	0,991	Excellent	Excellent
SN-Occ. Plane (Deg)	13,81±5,21	14,02±5,64	0,959	0,869	0,988	Excellent	Excellent
FMA (Deg)	25,56±6,19	25,24±6,48	0,971	0,908	0,992	Excellent	Excellent

Parameter	Measurement 1 (mean±SD)	Measurement 2 (mean±SD)	ICC	95% CI		Agreement (Cicchetti)	Agreement (Koo & Li)
N-ME (mm)	118,78±21,22	119,73±20,76	0,887	0,667	0,966	Excellent	Good
N-ANS (mm)	52,32±9,14	52,89±9,64	0,887	0,669	0,966	Excellent	Good
ANS-ME (mm)	66,46±12,69	66,84±11,78	0,897	0,695	0,969	Excellent	Good
S-GO (mm)	73,98±14,68	74,84±14,3	0,869	0,621	0,960	Excellent	Good
Z-ANGLE (Ang)	108,26±7,52	108,24±7,95	0,993	0,976	0,998	Excellent	Excellent
E-PLANE (mm)	-4±3,57	-3,92±3,72	0,989	0,965	0,997	Excellent	Excellent

22 Steiner

Parameter	Measurement 1 (mean±SD)	Measurement 2 (mean±SD)	ICC	95% CI		Agreement (Cicchetti)	Agreement (Koo & Li)
SNA (Deg)	82,79±2,42	82,93±2,51	0,922	0,763	0,977	Excellent	Excellent
SNB (Deg)	77,81±3,81	77,96±4,12	0,967	0,895	0,990	Excellent	Excellent
ANB (Deg)	4,97±2,84	4,96±2,84	0,999	0,997	1,000	Excellent	Excellent
I/ to NA (Deg)	18,65±4,82	18,9±3,88	0,925	0,772	0,978	Excellent	Excellent
I/ to NA (mm)	2,51±2,08	2,5±1,69	0,953	0,851	0,986	Excellent	Excellent
/I to NB (Deg)	21,73±8,41	36,67±45,54	0,000	-0,536	0,548	Poor	Poor
/I to NB (mm)	3,86±3,36	3,74±3,39	0,998	0,991	0,999	Excellent	Excellent
I/ to /I (Deg)	134,63±10,71	134,45±11,9	0,952	0,848	0,986	Excellent	Excellent
Occ to SN (Deg)	13,81±5,21	14,02±5,64	0,959	0,869	0,988	Excellent	Excellent
GOGN-SN (Deg)	36,2±7,84	36,1±7,9	0,992	0,975	0,998	Excellent	Excellent
POG to NB (mm)	1,82±1,75	1,82±1,82	0,991	0,971	0,997	Excellent	Excellent

23 Tweed

Parameter	Measurement 1 (mean±SD)	Measurement 2 (mean±SD)	ICC	95% CI		Agreement (Cicchetti)	Agreement (Koo & Li)
FMA (Deg)	25,56±6,19	25,24±6,48	0,971	0,908	0,992	Excellent	Excellent
FMIA (Deg)	64,63±10,3	65±11,1	0,965	0,888	0,990	Excellent	Excellent
IMPA (Deg)	87,71±7,77	87,6±9,68	0,951	0,846	0,985	Excellent	Excellent

24 Wits & Wits LS

Parameter	Measurement 1 (mean±SD)	Measurement 2 (mean±SD)	ICC	95% CI		Agreement (Cicchetti)	Agreement (Koo & Li)
Wits (mm)	3,72±3,99	3,53±4,78	0,944	0,826	0,983	Excellent	Excellent
Wits LS (mm)	3,72±3,99	3,53±4,78	0,944	0,826	0,983	Excellent	Excellent

25 Ricketts LS - MAX. POSITION

Parameter	Measurement 1 (mean±SD)	Measurement 2 (mean±SD)	ICC	95% CI		Agreement (Cicchetti)	Agreement (Koo & Li)
MAX DEPTH (Deg)	91,34±2,01	91,65±1,88	0,955	0,830	0,987	Excellent	Excellent
MAX HEIGHT (Deg)	57,44±3,45	57,53±3,81	0,980	0,935	0,994	Excellent	Excellent
SN TO PALATAL PLANE (Deg)	8,58±3,12	8,42±3	0,970	0,904	0,991	Excellent	Excellent

26 Ricketts LS - MAND. POSITION

Parameter	Measurement 1 (mean±SD)	Measurement 2 (mean±SD)	ICC	95% CI		Agreement (Cicchetti)	Agreement (Koo & Li)
FACIAL DEPTH (Deg)	87,18±3,18	87,46±3,24	0,982	0,936	0,995	Excellent	Excellent
FACIAL AXIS (Deg)	86,64±5,75	86,49±5,68	0,998	0,993	0,999	Excellent	Excellent
FACIAL TAPER (Deg)	68,35±4,03	68,46±4,78	0,938	0,809	0,982	Excellent	Excellent
MAND. PLANE (Deg)	25,56±6,19	25,24±6,48	0,971	0,908	0,992	Excellent	Excellent
CORPUS LENGTH (mm)	74,38±14,77	74,89±13,6	0,868	0,619	0,959	Excellent	Good
MAND. ARC (Deg)	26,54±5,92	25,89±4,99	0,905	0,716	0,971	Excellent	Excellent

27 Ricketts LS - MAX.TO MAND RELATIONSHIP

Parameter	Measurement 1 (mean±SD)	Measurement 2 (mean±SD)	ICC	95% CI		Agreement (Cicchetti)	Agreement (Koo & Li)
A pt. CONVEXITY (mm)	3,95±3,6	3,96±3,62	0,998	0,994	1,000	Excellent	Excellent
LOW.FACE.HEIGHT (Deg)	42,61±5,66	42,55±5,72	0,996	0,987	0,999	Excellent	Excellent

28 Ricketts LS - DENTURE RELATIONSHIP

Parameter	Measurement 1 (mean±SD)	Measurement 2 (mean±SD)	ICC	95% CI		Agreement (Cicchetti)	Agreement (Koo & Li)
MAX.1 to APo (mm)	5,78±3,16	5,76±2,85	0,979	0,932	0,994	Excellent	Excellent
MAX.6 to PTV (mm)	12,31±9,27	13,09±8,97	0,965	0,889	0,990	Excellent	Excellent
MAND. 1 to APo (mm)	0,18±2,45	0,08±2,58	0,985	0,951	0,996	Excellent	Excellent
HINGE AXIS ANGLE (Deg)	88,32±3,3	88,1±3,28	0,874	0,635	0,962	Excellent	Good
MAX.1 to MAND.1 (Deg)	134,63±10,71	134,45±11,9	0,952	0,848	0,986	Excellent	Excellent
OVERJET (mm)	4,88±2,57	4,93±2,41	0,991	0,972	0,997	Excellent	Excellent
OVERBITE (mm)	3,85±3,09	4,04±3,29	0,857	0,592	0,956	Excellent	Good

29 Ricketts LS - ESTHETICS

Parameter	Measurement 1 (mean±SD)	Measurement 2 (mean±SD)	ICC	95% CI		Agreement (Cicchetti)	Agreement (Koo & Li)
UPPER LIP to E-LINE (mm)	-3,41±3,7	-3,39±3,77	0,990	0,969	0,997	Excellent	Excellent
LOWER LIP to E-LINE (mm)	-4±3,57	-3,92±3,72	0,989	0,965	0,997	Excellent	Excellent

30 Steiner LS

Parameter	Measurement 1 (mean±SD)	Measurement 2 (mean±SD)	ICC	95% CI		Agreement (Cicchetti)	Agreement (Koo & Li)
SNA (Deg)	82,79±2,42	82,93±2,51	0,922	0,763	0,977	Excellent	Excellent
SNB (Deg)	77,81±3,81	77,96±4,12	0,967	0,895	0,990	Excellent	Excellent
ANB (Deg)	4,97±2,84	4,96±2,84	0,999	0,997	1,000	Excellent	Excellent
I/ to NA (Deg)	18,65±4,82	18,9±3,88	0,925	0,772	0,978	Excellent	Excellent
I/ to NA (mm)	2,51±2,08	2,5±1,69	0,953	0,851	0,986	Excellent	Excellent
/I to NB (Deg)	21,73±8,41	36,67±45,54	0,000	-0,536	0,548	Poor	Poor
/I to NB (mm)	3,86±3,36	3,74±3,39	0,998	0,991	0,999	Excellent	Excellent
I/ to /I (Deg)	134,63±10,71	134,45±11,9	0,952	0,848	0,986	Excellent	Excellent
Occ to SN (Deg)	13,81±5,21	14,02±5,64	0,959	0,869	0,988	Excellent	Excellent
GOGN-SN (Deg)	36,2±7,84	36,1±7,9	0,992	0,975	0,998	Excellent	Excellent
POG to NB (mm)	1,82±1,75	1,82±1,82	0,991	0,971	0,997	Excellent	Excellent

31 Tweed LS

Parameter	Measurement 1 (mean±SD)	Measurement 2 (mean±SD)	ICC	95% CI		Agreement (Cicchetti)	Agreement (Koo & Li)
FMA (Deg)	25,56±6,19	25,24±6,48	0,971	0,908	0,992	Excellent	Excellent
FMIA (Deg)	64,63±10,3	65±11,1	0,965	0,888	0,990	Excellent	Excellent
IMPA (Deg)	87,71±7,77	87,6±9,68	0,951	0,846	0,985	Excellent	Excellent

32 SLU LS

Parameter	Measurement 1 (mean±SD)	Measurement 2 (mean±SD)	ICC	95% CI		Agreement (Cicchetti)	Agreement (Koo & Li)
SNA (Deg)	82,79±2,42	82,93±2,51	0,922	0,763	0,977	Excellent	Excellent
PNS-A (Deg)	49,2±8,75	49,56±8,26	0,899	0,701	0,970	Excellent	Good
PTV-6 (mm)	12,31±9,27	13,09±8,97	0,965	0,889	0,990	Excellent	Excellent
UI-SN (Deg)	101,44±5,98	101,83±5,33	0,963	0,881	0,989	Excellent	Excellent
UI-NA (Deg)	18,65±4,82	18,9±3,88	0,925	0,772	0,978	Excellent	Excellent
UI-NA (mm)	2,51±2,08	2,5±1,69	0,953	0,851	0,986	Excellent	Excellent
SNB (Deg)	77,81±3,81	77,96±4,12	0,967	0,895	0,990	Excellent	Excellent
NS-Gn (Deg)	69,04±5,57	69,1±5,59	0,997	0,989	0,999	Excellent	Excellent
S-Ar (mm)	35,38±7,29	35,88±7,87	0,909	0,727	0,973	Excellent	Excellent
AR-Gn (mm)	105,1±20,91	105,89±20,12	0,896	0,692	0,969	Excellent	Good
POG-NS (mm)	1,82±1,75	1,82±1,82	0,991	0,971	0,997	Excellent	Excellent
IMPA (Ang)	87,71±7,77	87,6±9,68	0,951	0,846	0,985	Excellent	Excellent
LI-APOG (mm)	0,18±2,45	0,08±2,58	0,985	0,951	0,996	Excellent	Excellent
LI-NB (Ang)	21,73±8,41	36,67±45,54	0,000	-0,536	0,548	Poor	Poor
LI-NB (mm)	3,86±3,36	3,74±3,39	0,998	0,991	0,999	Excellent	Excellent
FMIA (Ang)	64,63±10,3	65±11,1	0,965	0,888	0,990	Excellent	Excellent
LI/UI (Deg)	134,63±10,71	134,45±11,9	0,952	0,848	0,986	Excellent	Excellent
ANB (Ang)	4,97±2,84	4,96±2,84	0,999	0,997	1,000	Excellent	Excellent
WITS (mm)	3,72±3,99	3,53±4,78	0,944	0,826	0,983	Excellent	Excellent
SN-PAL.PLANE (Deg)	8,58±3,12	8,42±3	0,970	0,904	0,991	Excellent	Excellent
SN-Occ. Plane (Deg)	13,81±5,21	14,02±5,64	0,959	0,869	0,988	Excellent	Excellent
FMA (Deg)	25,56±6,19	25,24±6,48	0,971	0,908	0,992	Excellent	Excellent
N-ME (mm)	118,78±21,22	119,73±20,76	0,887	0,667	0,966	Excellent	Good
N-ANS (mm)	52,32±9,14	52,89±9,64	0,887	0,669	0,966	Excellent	Good
ANS-ME (mm)	66,46±12,69	66,84±11,78	0,897	0,695	0,969	Excellent	Good

Parameter	Measurement 1 (mean±SD)	Measurement 2 (mean±SD)	ICC	95% CI		Agreement (Cicchetti)	Agreement (Koo & Li)
S-GO (mm)	73,98±14,68	74,84±14,3	0,869	0,621	0,960	Excellent	Good
Z-ANGLE (Ang)	108,26±7,52	108,24±7,95	0,993	0,976	0,998	Excellent	Excellent
E-PLANE (mm)	-4±3,57	-3,92±3,72	0,989	0,965	0,997	Excellent	Excellent

33 Downs LS - SKELETAL

Parameter	Measurement 1 (mean±SD)	Measurement 2 (mean±SD)	ICC	95% CI		Agreement (Cicchetti)	Agreement (Koo & Li)
FACIAL ANGLE (Deg)	87,18±3,18	87,46±3,24	0,982	0,936	0,995	Excellent	Excellent
ANGLE CONVEXITY (DOWNS) (Deg)	172,18±7,9	172,09±8,03	0,999	0,996	1,000	Excellent	Excellent
A-B PLANE (Deg)	7,55±2,79	7,53±2,73	0,998	0,993	0,999	Excellent	Excellent
MAND. PLANE (Deg)	25,56±6,19	25,24±6,48	0,971	0,908	0,992	Excellent	Excellent
Y AXIS (Deg)	60,48±4,03	60,39±4,07	0,987	0,957	0,996	Excellent	Excellent

34 Downs LS - DENTAL

Parameter	Measurement 1 (mean±SD)	Measurement 2 (mean±SD)	ICC	95% CI		Agreement (Cicchetti)	Agreement (Koo & Li)
OCCLUSAL PLANE (Deg)	20,25±49,62	20,3±49,66	0,999	0,997	1,000	Excellent	Excellent
UI to LI (Deg)	134,63±10,71	134,45±11,9	0,952	0,848	0,986	Excellent	Excellent
LI to Occ PL. (Deg)	69,89±10,12	70,31±12,43	0,966	0,890	0,990	Excellent	Excellent
LI to MAND (Deg)	87,71±7,77	87,6±9,68	0,951	0,846	0,985	Excellent	Excellent
UI to A-Pog (mm)	5,78±3,16	5,76±2,85	0,979	0,932	0,994	Excellent	Excellent

35 Jarabak LS

Parameter	Measurement 1 (mean±SD)	Measurement 2 (mean±SD)	ICC	95% CI		Agreement (Cicchetti)	Agreement (Koo & Li)
SADDLE ANGLE (Deg)	123,77±3,4	123,92±3,71	0,967	0,895	0,990	Excellent	Excellent
ARTICULAR ANGLE (Deg)	144,73±5,17	144,55±5,65	0,955	0,858	0,987	Excellent	Excellent

Parameter	Measurement 1 (mean±SD)	Measurement 2 (mean±SD)	ICC	95% CI		Agreement (Cicchetti)	Agreement (Koo & Li)
GONIAL ANGLE (Deg)	125,61±4,8	125,48±5,2	0,945	0,828	0,984	Excellent	Excellent
SUM OF ANGLES (Deg)	394,11±7,67	393,94±7,8	0,992	0,972	0,998	Excellent	Excellent
UPPER GONIAL ANGLE (Deg)	52,07±3,84	51,94±4	0,946	0,831	0,984	Excellent	Excellent
LOWER GONIAL ANGLE (Deg)	73,54±6,68	73,53±6,71	0,997	0,991	0,999	Excellent	Excellent
ANT. CRANIAL BASE (mm)	68,34±12,44	68,61±12,4	0,902	0,706	0,970	Excellent	Excellent
POST. CRANIAL BASE (mm)	35,38±7,29	35,88±7,87	0,909	0,727	0,973	Excellent	Excellent
RAMUS HEIGHT (mm)	42,31±8,8	42,75±7,67	0,837	0,544	0,949	Excellent	Good
MANDIBULAR BODY (mm)	74,77±15,29	75,29±15,55	0,897	0,696	0,969	Excellent	Good
POST. FACE HEIGHT (mm)	73,98±14,68	74,84±14,3	0,869	0,621	0,960	Excellent	Good
ANT. FACE HEIGHTL (mm)	115,76±21,33	116,68±20,6	0,893	0,684	0,968	Excellent	Good
PFH:AFH (%)	63,45±4,28	63,69±4,4	0,958	0,867	0,988	Excellent	Excellent
ACB:MAND.BODY (%)	0,91±0,06	0,91±0,09	0,850	0,576	0,954	Excellent	Good
UI to SN (Deg)	101,44±5,98	101,83±5,33	0,963	0,881	0,989	Excellent	Excellent
UI to FH (Deg)	110±4,8	110,55±4,08	0,923	0,764	0,977	Excellent	Excellent
UPPER FACE HEIGHT (%)	45,08±2,02	45±1,95	0,989	0,965	0,997	Excellent	Excellent
LOWER FACE HEIGHT (%)	55,89±2,13	55,85±2,13	0,999	0,995	1,000	Excellent	Excellent
